# Emergence of Colistin Resistance Gene *mcr*-*10* in *Enterobacterales* Isolates Recovered from Fecal Samples of Chickens, Slaughterhouse Workers, and a Nearby Resident

**DOI:** 10.1128/spectrum.00418-22

**Published:** 2022-04-12

**Authors:** Linna Xu, Fen Wan, Hao Fu, Biao Tang, Zhi Ruan, Yonghong Xiao, Qixia Luo

**Affiliations:** a State Key Laboratory for Diagnosis and Treatment of Infectious Diseases, Collaborative Innovation Center for Diagnosis and Treatment of Infectious Diseases, the First Affiliated Hospital of Medical School, College of Medicine, Zhejiang University, Hangzhou, China; b School of Laboratory Medicine and Biotechnology, Hangzhou Medical College, Hangzhou, China; c Institute of Agro-product Safety and Nutrition, Zhejiang Academy of Agricultural Sciences, Hangzhou, China; d Department of Clinical Laboratory, Sir Run Run Shaw Hospital, Zhejiang University School of Medicine, Hangzhou, China; e Jinan Microecological Biomedicine Shandong Laboratory, Jinan, China; University of Guelph

**Keywords:** polymyxins, antibiotic resistance, *mcr*-*10*, *Enterobacterales*, one health

## Abstract

The wide spread of plasmid-borne mobilized colistin resistance (*mcr*) genes from animals to humans broadly challenges the clinical use of polymyxins. Here, we evaluated the incidence of a recently reported *mcr* variant, *mcr*-*10*, in animals and humans in the same area. Our results revealed the presence of novel *mcr-10*-carrying plasmids in two Klebsiella pneumoniae isolates from chickens, one Escherichia coli isolate from slaughterhouse workers, and a chromosome-borne *mcr-10* gene in Enterobacter kobei from a healthy resident in the same region. It is worth mentioning that the multidrug-resistant ST11 K. pneumoniae isolates coharboring *mcr*-*10* and *mcr-8* genes in two separate plasmids not only were resistant to polymyxins (MIC = 8 mg/L) but also showed reduced susceptibility to tigecycline (MIC ≥ 2 mg/L) due to the *tet*(A) mutation or the *tmexCD1-toprJ1* gene cluster. The structure *xerC*-*mcr10*-*insCinsD*-like was found in genetic environments of both the plasmid and chromosome carrying *mcr-10*. We compared genomic epidemiological characteristics of *mcr-10*-harboring bacteria available in 941,449 genomes in the NCBI database (including strains of K. pneumoniae, E. coli, and *E. kobei*) with isolates in this study. The results indicated a sporadic distribution of *mcr-10* all around the world and in a variety of sources, including humans, environments, and animals, which confirms that *mcr-10* has spread among various hosts and warrants close monitoring and further future studies.

**IMPORTANCE** We discovered *mcr-10*-harboring isolates in the “one health” approach and reported for the first time multidrug-resistant clinically threatening ST11 K. pneumoniae isolates coharboring *mcr*-*10* and *mcr-8* genes that are resistant to polymyxins and show reduced susceptibility to tigecycline. The exhaustive screening of 941,449 bacterial genomes in the GenBank database discovered a sporadic distribution of *mcr-10*-harboring isolates all around the world in a variety of sources, especially humans, which warrants close monitoring and a particular concern in clinical settings.

## INTRODUCTION

The inappropriate use of antibiotics exerts selective pressure on bacterial pathogens and commensal microbes, which favors the emergence of multidrug-resistant (MDR) superbugs (1, 2). *Enterobacterales* are the most common pathogens or commensal organisms spread between humans, animals, and the environment. Colistin (polymyxin E) is one of the last-resort antimicrobial agents for multidrug-resistant (MDR) *Enterobacterales*, such as carbapenem-resistant *Enterobacterales* (CRE) (3, 4). However, the overuse and misuse of colistin in the livestock industry and clinics have led to the global emergence of colistin-resistant pathogens (5, 6). *Enterobacterales* strains with acquired colistin resistance have emerged worldwide, significantly jeopardizing the efficacy of colistin (7–9). Mobile colistin resistance genes (*mcr*) have become a major mechanism mediating decreased colistin susceptibility in *Enterobacterales* and pose a serious challenge to public health, the livestock industry, and the environment.

Liu et al. (10) first reported the emergence of *mcr-1* in *Enterobacterales* isolates of human and animal origin in China, and in the past 5 years, 10 major *mcr* variants (*mcr*-*1* to -*10*) have been reported worldwide (11–19). However, higher prevalence rates of *mcr-1*-positve isolates are observed in Asian countries, especially in food animals and farm environments, which can be caused by long-term exposure to high colistin concentrations (5, 20, 21). In addition, multiple *mcr* variants, *mcr*-*2* to -*10*, have been occasionally reported and are not as widely disseminated as *mcr-1*. Recently, Wang et al. (19) identified *mcr-10* on an IncFIA plasmid of an Enterobacter roggenkampii clinical strain, which has the highest nucleotide identity with *mcr-9* and confers a 4-fold increase in colistin MIC. Following this report, studies reported *mcr-10*-harboring Enterobacter spp. isolated from animal and hospital sewage water and Cronobacter sakazakii isolated from a healthy person (22–24). Since it was a newly discovered *mcr* variant, case reports about *mcr-10* were still rare.

This study discovered plasmid-borne *mcr*-*10*-harboring Klebsiella pneumoniae from chickens, Escherichia coli from slaughterhouse workers, and Enterobacter kobei from a nearby healthy resident. It is worth mentioning that the K. pneumoniae isolates were multidrug-resistant ST11 isolates that coharbored *mcr*-*10* and *mcr-8* on two separate plasmids, had a reduced susceptibility to tigecycline, and had several virulence genes. We extensively screened the *mcr-10*-carrying isolates in the NCBI database and compared their genomes and plasmids with the isolates in our study. To our knowledge, this is the first detailed report of *mcr-10-*harboring plasmids from K. pneumoniae and E. coli and various *mcr-10-*harboring *Enterobacterales* in the “one health” approach. Our results suggest that *mcr-10* has spread among humans and animals, which requires increasing efforts to closely monitor the emergence of more resistant isolates and further studies to investigate the current situation.

## RESULTS

### Identification of *mcr-10* in *Enterobacterales* isolates and transferability of the *mcr-10*-harboring plasmids.

To study the spread and transmission of *mcr-10* between animals and humans, we chose the rural area near Hangzhou, China, where there are a chicken slaughterhouse and villages nearby. Fecal samples of chickens, slaughterhouse workers, and healthy people in villages were collected. A total of 200 *Enterobacterales* strains were isolated from fecal samples in this study, including 102 from chickens, 58 from slaughterhouse workers, and 40 from nearby residents. PCR was conducted to screen *mcr-10* using primers as described in Materials and Methods. Four *mcr-10*-positive isolates were obtained, including two K. pneumoniae isolates (KP46 and KP57) from chickens, one E. coli isolate (EC81) from a slaughterhouse worker, and one *E. kobei* isolate (EK6) from a nearby resident. PCR and Sanger sequencing demonstrated that these four *mcr-10* genes are all *mcr-10.1* (GenBank accession number MN179494.1). KP46 and KP57 had low-level resistance to polymyxins (MIC = 8 mg/L), and EK6 had high-level resistance to polymyxins (MIC = 128 mg/L), while EC81 was susceptible to polymyxins (MIC = 2 mg/L) but close to the breakpoint defined by EUCAST (Table 1).

**TABLE 1:** Antimicrobial susceptibility profiles of *mcr-10*-carrying isolates and their transconjugants

Strain	MIC (mg/L) of drug^*a*^:																	
	AMX	CZO	MOX	LVX	ATM	CRO	FEP	AMK	CZA	CAZ	CSL	ETP	CIP	MEM	IPM	TGC	PMB	CST
KP46	>128	>128	1	>32	>64	>64	16	>128	0.5	64	32	0.125	>32	0.03	0.5	8	8	8
KP46/J53	8	2	0.25	0.06	0.125	0.06	0.03	2	0.125	0.25	≤0.125	≤0.004	0.015	0.06	0.5	4	4	4
KP57	>128	>128	2	>32	>64	>64	16	2	0.5	64	64	0.125	>32	0.06	0.25	2	8	8
KP57/J53	8	2	0.25	0.06	0.125	0.125	0.03	2	0.125	0.25	≤0.125	≤0.004	0.015	0.03	0.5	2	4	4
EK6	8	4	0.25	0.06	0.06	0.125	0.03	4	0.125	0.25	0.5	≤0.004	0.03	0.015	0.5	1	128	128
EC81	8	≤1	0.25	0.06	≤0.06	0.06	≤0.015	4	≤0.06	≤0.06	≤0.125	≤0.004	0.015	≤0.008	0.25	1	2	2
J53	16	2	0.5	0.03	0.25	0.125	0.03	2	0.125	0.25	≤0.125	≤0.004	0.015	0.015	0.5	0.25	0.5	1

a AMX, amoxicillin; CZO, cefazolin; MOX, latamoxef; LVX, levofloxacin; ATM, aztreonam; CRO, ceftriaxone; FEP, cefepime; AMK, amikacin; CZA, ceftazidime-avibactam; CAZ, ceftazidime; CSL, cefoperazone-sulbactam; ETP, ertapenem; CIP, ciprofloxacin; MEM, meropenem; IPM, imipenem; TGC, tigecycline; PMB, polymyxin B; CST, colistin.

Antimicrobial susceptibility analysis of these four *mcr-10*-harboring isolates indicated that K. pneumoniae isolates from chickens were resistant to most antimicrobial agents, including most cephalosporins and one of the clinical last-resort antibiotics, tigecycline (MIC = 8 mg/L and 2 mg/L, respectively), but remained susceptible to the carbapenems fosfomycin and moxalactam (Table 1). However, EK6 and EC81, the isolates from humans, were susceptible to almost all the tested antibiotics (Table 1).

Conjugation experiments showed that isolates KP46 and KP57 could partially transfer their colistin resistance phenotypes to E. coli J53, while EK6 and EC81 could not. The conjugants of KP46 and KP57 did not contain *mcr-10*, which meant that *mcr-10* was not transferable in both isolates. However, the colistin-resistant conjugants were both harboring *mcr-8* genes that were transferred from plasmids in both KP46 and KP57. S1-PFGE (pulsed-field gel electrophoresis) and Southern hybridization showed that KP46 and KP57 carried five and four plasmids, respectively, on which the *mcr-10* gene was located at both ∼173.4 kb and ∼216.9 kb while the *mcr-8* gene was located at both ∼78.2 kb and ∼104.5 kb on plasmids (see Fig. S1 in the supplemental material). EC81 carried two plasmids, and the *mcr-10* gene was located between ∼54.7 kb and ∼78.2 kb (Fig. S1). EK6 did not have the *mcr-10-*carrying plasmids, which indicates that *mcr-10* might be found on the chromosome.

### Genome analysis of the four *mcr-10*-harboring isolates in this study.

Complete genomes of these four isolates were analyzed. *In silico* multilocus sequence typing (MLST) analysis revealed that KP46 and KP57 both belonged to ST11, the dominant pan-drug-resistant sequence type widely disseminated in China, which causes an infection that is challenging to cure. EK6 belongs to ST1605, and EC81 belongs to ST216.

KP46 consists of a 5,253,530-bp chromosome and five plasmids (pKP46-mcr10, pKP46-mcr8, pKP46-3, pKP46-4, and pKP46-5) (Table 2). KP57 consists of a 5,255,236-bp chromosome and four plasmids (pKP57-mcr10, pKP57-mcr8, pKP57-3, and pKP57-4) (Table 2). Resistance determinants *fosA*, *oqxA*, *oqxB*, and *bla*_SHV-182_ were detected on the chromosomes of both KP46 and KP57. pKP57-mcr10, pKP57-mcr8, pKP57-3, and pKP57-4 had 99.9% identity (100% coverage) to pKP46-mcr10, pKP46-mcr8, pKP46-4, and pKP46-5, respectively. Six resistance genes were carried on pKP57-mcr10 including *mcr-10*, *qnrB52*, *sul1*, *tet*(A), *bla*_TEM-1B_, and *floR*, which confer multidrug resistance. Only one antimicrobial resistance gene, *mcr-8*, was identified on both pKP57-mcr8 and pKP46-mcr8. Other resistance genes such as *bla*_CTX-M-15_ and *qnrS1* were found on the other two plasmids (Table 2). Considering there were only 56 core-genome single nucleotide polymorphisms (SNPs) of KP46 compared with KP57, and four plasmids were almost the same, KP46 might have evolved from a KP57-like strain that gained the big plasmid pKP46-3 or KP46 lost pKP46-3 to produce KP57. EC81 consists of a 4,785,944-bp chromosome and two plasmids (pEC81-mcr10 and pEC81-2); no other resistance genes were identified in pEC81-mcr10, except *mcr-10* (Table 2). EK6 consists of a 4,860,688-bp chromosome that contains resistance genes *mcr-10* and *bla*_ATC-9_, and no plasmids were identified (Table 2).

**TABLE 2:** Features of the isolates

Isolate	Source	*mcr* type(s)	DNA type	DNA size (bp)	Resistance gene(s)	MLST/plasmid replicon	Accession no.
KP46	Chicken	*mcr-10*, *mcr-8*	Chromosome	5,253,530	*fosA*, *oqxA*, *oqxB*, *bla*_SHV-182_	ST11	CP088120
			pKP46-mcr10	185,056	*mcr-10*, *qnrB52*, *sul1*, *tet*(A), *bla*_TEM-1B_, *floR*	IncFIB, IncFII	CP088121
			pKP46-mcr8	101,184	*mcr-8*	IncFIA, IncFII	CP088122
			pKP46-3	272,060	*msr(E)*, *aph(3′)-Ib*, *aadA1*, *aadA2b*, *armA*, *aph(6)-Id*, *aph(3′)-Ia*, *qnrB4*, *sul1*, *sul3*, *bla*_DHA-1_, *mph(E)*, *qacE*, *cmlA1*	IncFIB, IncHI1B	CP088123
			pKP46-4	72,636	*qnrS1*, *bla*_CTX-M-15_, *bla*_TEM-1B_	—^*a*^	CP088124
			pKP46-5	82,962	*aph(3′)-Ia*, *aph(3′)-Ib*, *aph(3′)-IId*, *aadA16*, *aph(6)-Id*, *aph(6)-Id-cr*, *tet*(A), *mph(A)*, *aac(6′)-Ib-cr*, *sul1*, *sul2*, *afrA27*, *arr-3*, *qacE*	IncFIA, IncQ1, IncR	CP088125

KP57	Chicken	*mcr-10*, *mcr-8*	Chromosome	5,255,236	*fosA*, *oqxA*, *oqxB*, *bla*_SHV-182_	ST11	CP088126
			pKP57-mcr10	186,040	*mcr-10*, *qnrB52*, *tet*(A), *bla*_TEM-1B_, *floR*	IncFIB, IncFII	CP088127
			pKP57-mcr8	100,928	*mcr-8*	IncFIA, IncFII	CP088128
			pKP57-3	83,070	*aph(3)-lb*, *aph(6)-lb*, *aadA16*, *aac(3)-IId*, *aac(6)-Ib-cr*, *arr-3*, *mph(A)*, *dfrA27*, *sul1*, *sul2*, *tet*(A), *qacE*	IncFIA, IncQ1, IncR	CP088129
			pKP57-4	72,637	*qnrS1*, *bla*_CTX-M-15_, *bla*_TEM-1B_	—	CP088130

EC81	Human	*mcr-10*	Chromosome	4,785,944	—	ST216	CP088131
			pEC81-mcr10	62,662	*mcr-10*	IncFIA	CP088132
			pEC81-2	104,151	—	IncY	CP088133

EK6	Human	*mcr-10*	Chromosome	4,860,688	*mcr-10*, *bla*_ACT-9_	ST1605	CP088119

a —, no resistance genes or plasmid replicon was found.

The *mrk* gene cluster (*mrkABCDF*), encoding type 3 fimbrial adhesins, which mediate adhesion to the surface of endothelial cells and are usually found in K. pneumoniae clinical isolates, was present on the pKP46-mcr10 and pKP57-mcr10 plasmids. The *mrk* cluster remained truncated in pEC-mcr10 lacking *mrkD*. Serum complement killing assay and Galleria mellonella infection experiments indicated an increased virulence of EC81 compared with E. coli ATCC 25922 but no significantly increased virulence of KP46, KP57, and EK6 (Fig. S2).

We found two amino acid mutations in EK6 PmrB (Q168P and N233T) compared with the reference strain Enterobacter kobei UCI-24 but did not find mutations in PmrA and PhoPQ. Q168P is in the His kinase A (phosphoacceptor) domain, which is very important for the function of PmrB.

### Features of *mcr-10*-harboring isolates and plasmids.

**FIG 1:**
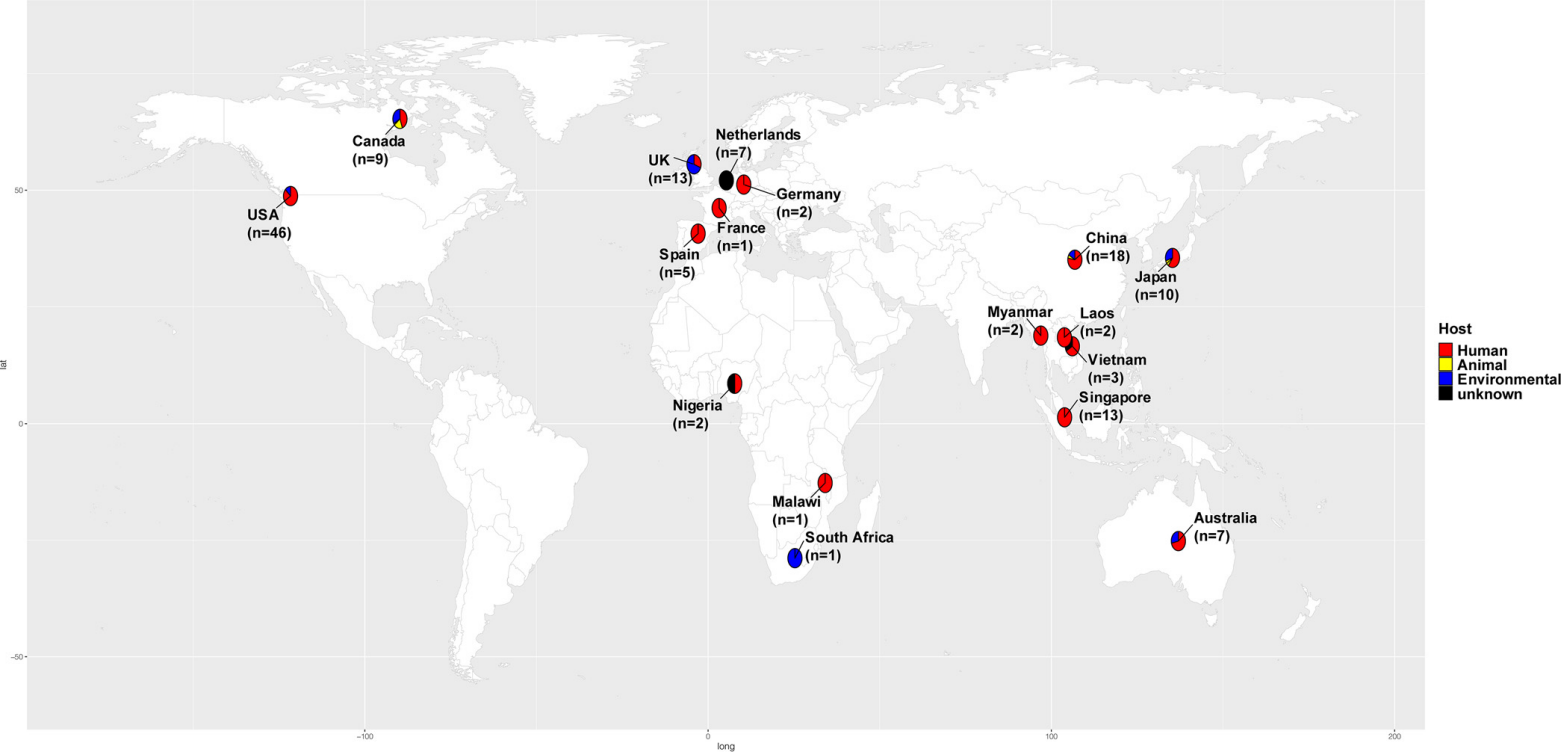
Geographical distribution of the 142 isolates with definitive locations, among all the 151 *mcr-10*-harboring isolates from the NCBI database (see Table S1 in the supplemental material). Different hosts of the isolates are labeled with different colors. Red, yellow, blue, and black indicate human, animal, environmental, and unknown sources, respectively. The number of all the *mcr-10*-harboring isolates in each country is labeled on the world map under the country name. The world map was created using the corresponding map data with R package ggplot2 v3.3.5 (https://github.com/tidyverse/ggplot2).

A total of 151 *mcr-10*-harboring isolates were screened out from 941,449 genomes of the GenBank database (Table S1), including 15 species of 7 genera (*Citrobacter*, *Cronobacter*, Enterobacter, Escherichia, Klebsiella, *Kluyvera*, and *Raoultella*) of the family *Enterobacterales*, distributed in 17 countries on five continents. Of the strains with a clear origin, most of the strains were from humans (101/132), and the next most common were from water (24/132); only a few were from animals or animal food (6/132). Of the strains with clear locations, the top six were from the United States, the United Kingdom, Singapore, China, Canada, and Japan (Fig. 1; see also Table S1).

**FIG 2:**
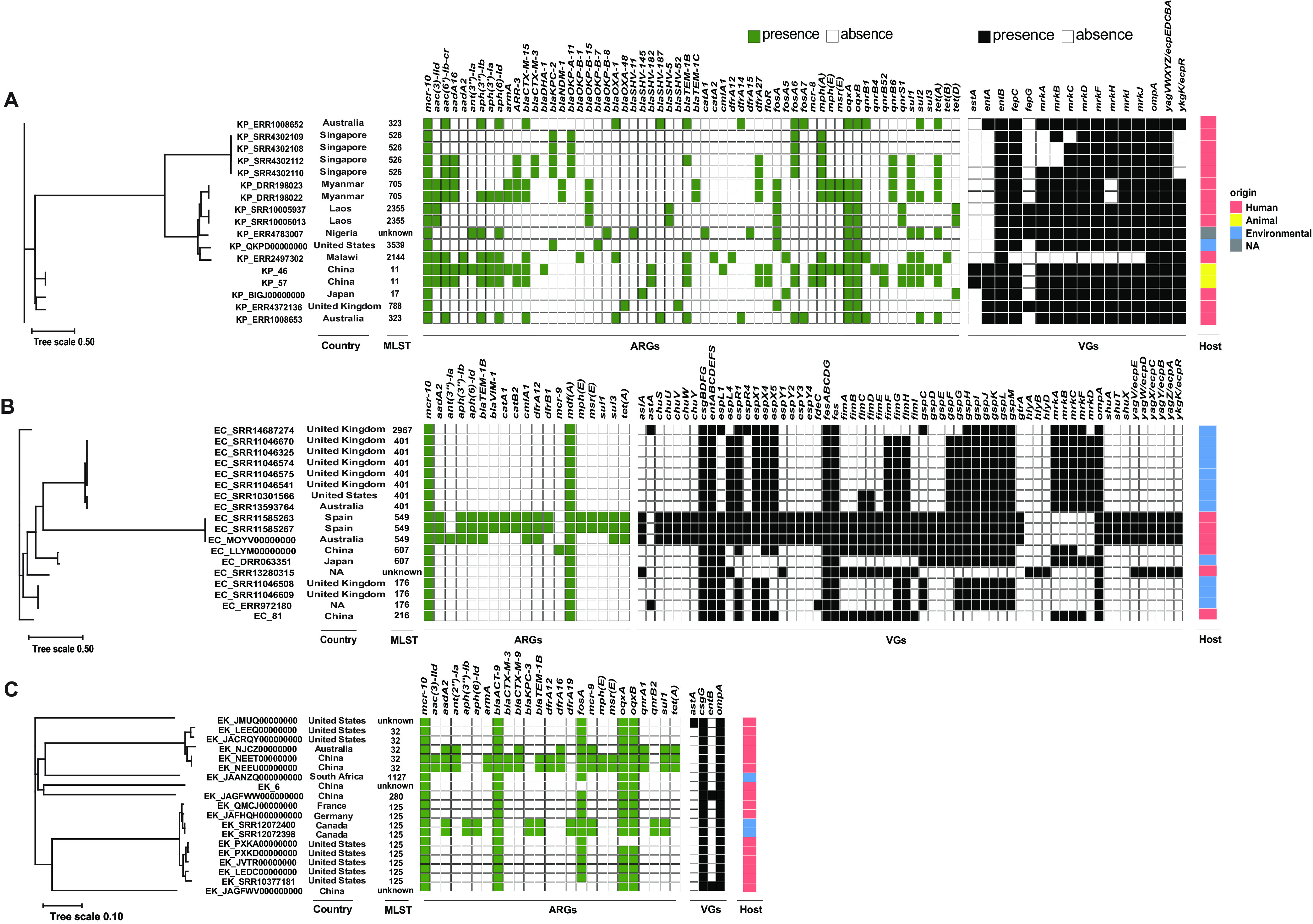
Phylogenetic tree generated from the core-genome sequences of the *mcr-10*-harboring isolates identified in this study and other *mcr-10*-harboring isolates within the same species from the GenBank database. The sample identifier (ID), location, MLST, and sources are indicated for each isolate. Significant antimicrobial resistance genes (ARGs) and major virulence-associated genes (VGs) are shown. (A) Phylogenetic tree and distribution of *mcr-10*-positive K. pneumoniae isolates; (B) phylogenetic tree and distribution of *mcr-10*-positive E. coli isolates; (C) phylogenetic tree and distribution of *mcr-10*-positive Enterobacter kobei isolates. NA, not available.

Phylogenetic comparison of the *mcr-10*-positive isolates in this study with their corresponding *mcr-10*-positive species from the GenBank database was performed with categories of phylogroups, location, multilocus sequencing types, antimicrobial resistance genes, virulence-associated genes, and sources (Fig. 2A to C). The results indicated a sporadic distribution of *mcr-10* worldwide and a broad spectrum of sequence types. There was no animal source of *mcr-10*-harboring K. pneumoniae from the database except for two isolates from this study (Fig. 2A). Most of the *mcr-10*-harboring E. coli isolates were isolated from the environment (water) located in the United Kingdom, but that might have been because of the sample bias (Fig. 2B). ST125 accounted for 47.4% (9/19) of all *mcr-10*-harboring *E. kobei* isolates (Fig. 2C).

**FIG 3:**
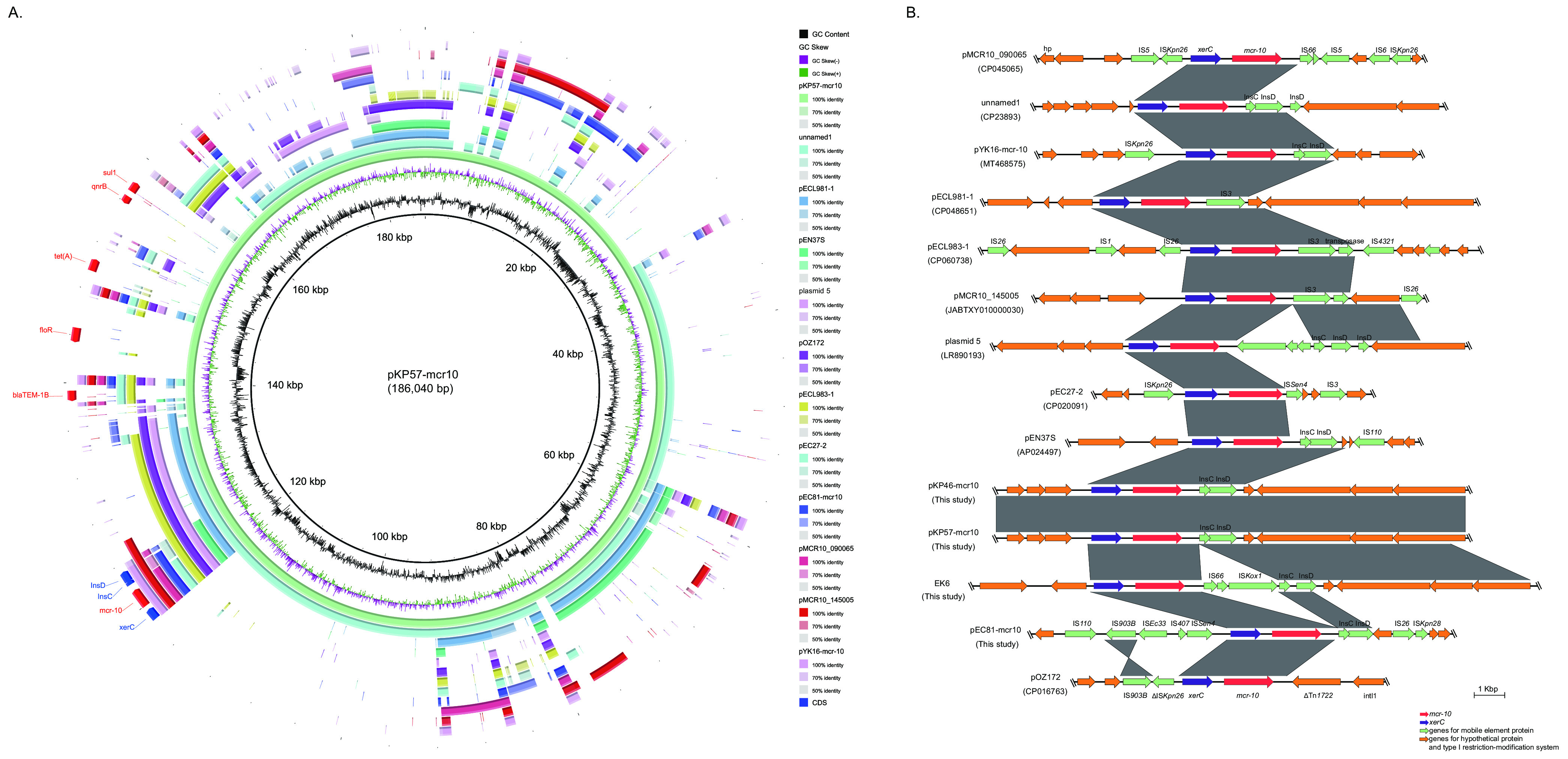
Genetic organization of plasmid harboring *mcr-10*. (A) BLASTN comparison of the complement sequences of the *mcr-10* plasmid found in this study and those deposited in the GenBank database. (B) Schematic representation and comparison of the genetic environments of the *mcr-10*-flanking region in each genomic backbone type. Arrows indicate the direction of transcription of each gene, and different genes are shown in different colors. Regions of 90.0% nucleotide sequence identity are shaded gray.

BLAST searching in GenBank suggested that the three *mcr-10*-bearing plasmids were novel, and replicon typing showed that they belong to IncFIB-FII (pKP46-mcr10 and pKP57-mcr10) and IncFIA (pEC81-mcr10). No genes encoding conjugation-related proteins were found in pKP46-mcr10, pKP57-mcr10, or pEC81-mcr10, suggesting that the plasmid is non-self-transmissible, which was consistent with the results of the conjugation experiment. pKP57-mcr10 and pEC81-mcr10 shared only 18% coverage, showing that they were from different plasmids with no direct transmission relationship. Comparing the three plasmids with the only 10 *mcr-10*-harboring completely sequenced plasmids in GenBank, we found a diversity of genes in the plasmids (Fig. 3A) but a relatively undiversified plasmid replicon type which most likely belongs to IncF (Table S2). This study did not find any complete site-specific recombination sites in *mcr-10*-harboring plasmids.

**FIG 4:**
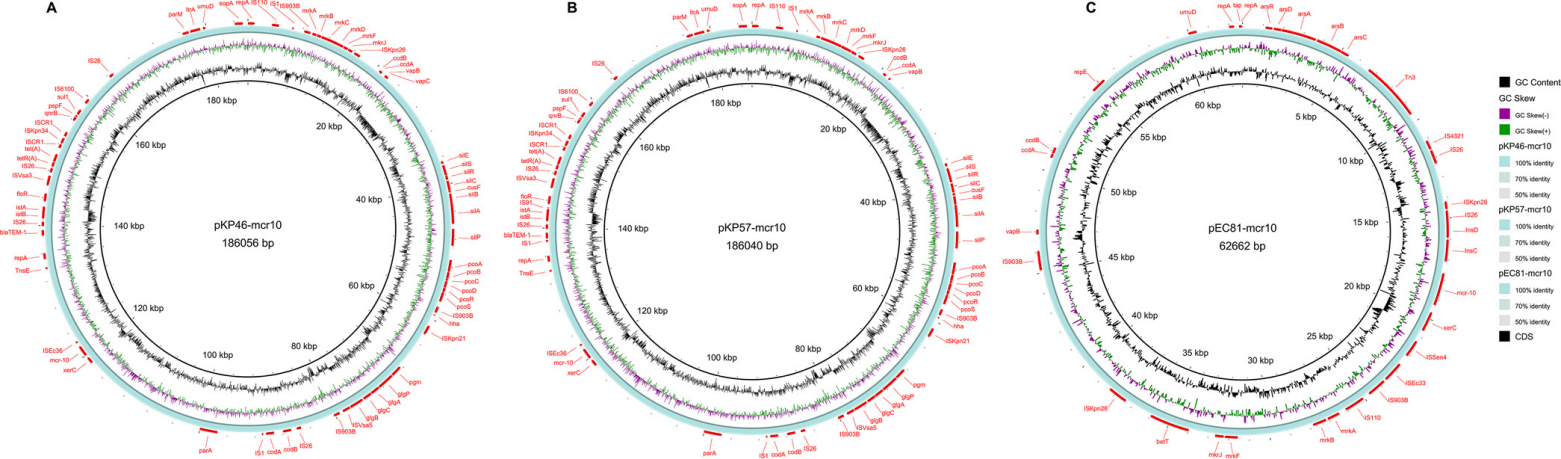
Structure of plasmid carrying *mcr-10* from strains originating from Shaoxing in this study. (A) Structure of plasmid pKP46-mcr10 from K. pneumoniae strain KP46. (B) Structure of plasmid pKP57-mcr10 from K. pneumoniae strain KP57. (C) Structure of plasmid pEC81-mcr10 from E. coli strain EC81. The GC content, GC skew, nucleotide identity, and coding sequences (CDS) are all labeled in corresponding colors, and the genes are shown by the red arrow around the plasmid.

In all *mcr-10*-positive plasmids (Fig. 3 and Table S2), the tyrosine site-specific recombinase gene *xerC*, which mediates the mobilization of genetic elements, was found upstream of all the plasmids, as well as the *insCinsD-*like region (Fig. 3B). The ∼6-kb *xerC-mcr10-insCinsD-*like gene arrangement is quite well conserved through the spreading process of *mcr*-*10*-mediated colistin resistance among Enterobacter species, whereas IS*Ec36* transposase InsC was identified downstream in all three isolates in this study but in only 3/10 plasmids from the GenBank database, as *insC* and *insD* were not complete genes in some of the plasmids (Fig. 3B). Moreover, we also performed synteny analysis with the *mcr-10* gene detected on the chromosome genome of EK6. The result was the same as that of *xerC* located upstream of *mcr*-*10* (Fig. 3B). The structure of the three *mcr-10*-positive plasmids is detailed in Fig. 4. The virulence gene *mrkABCDFJ* cluster was closely arranged in KP46 and KP57, but for EC81, only *mrkA*, *mrkB*, *mrkF*, and *mrkJ* were present (Fig. 4).

### Genetic features of *mcr-8*-carrying and other plasmids in KP46 and KP57.

**FIG 5:**
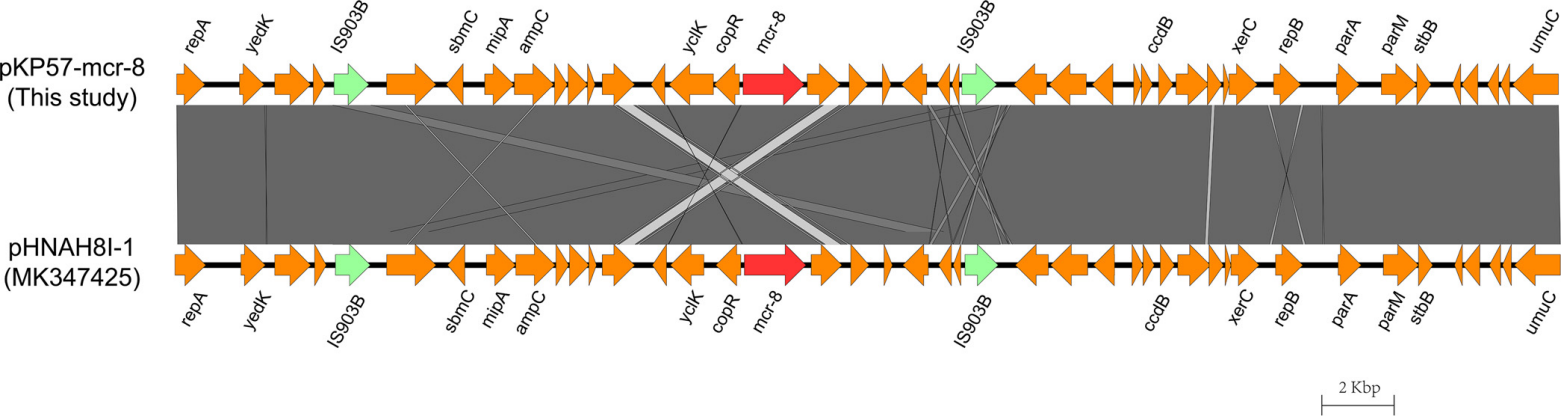
Schematic representation and comparison of the genetic environments of the *mcr-8*-flanking region in pKP57-mcr8 and pHNAH81-1 (accession number MK347425). Arrows indicate the direction of transcription of each gene, and different genes are shown in different colors. Regions of 90.0% nucleotide sequence identity are shaded gray.

The transconjugation assay showed that pKP46-mcr8 and pKP57-mcr8 were self-transferable, and the frequency of plasmid transfer was 10^−6^ per recipient cell. The plasmid replicon type of pKP57-mcr8 was identified as multireplicon plasmids with IncFIA-FII replicons (Table 2). Sequences of pKP46-mcr8 and pKP57-mcr8 displayed high identity (99.79%) by BLASTN. The genetic environment of the *mcr-8*-flanking region of pKP57-mcr8 was >90% identical to pHNAH81-1 (accession number MK347425), a representative plasmid-encoded resistance-nodulation-division (RND) efflux pump conferring resistance to multiple drugs in K. pneumoniae (Fig. 5). Phylogenetic analysis and gene context comparison revealed that *mcr-8* was flanked by complete insertion sequence IS*903B* (Fig. 5). This increased the risk for the transposition and dissemination of pKP46-mcr8 and pKP57-mcr8.

We also noticed that KP46 showed the highest resistance level to tigecycline, up to 8 mg/L. After screening for the tigecycline resistance genes, we identified the newly reported resistance-nodulation-division (RND) efflux pump gene cluster *tnfxB1-tmexCD1-toprJ1* located on plasmid pKP46-3, an IncFIB-HI1B plasmid containing multiple resistance genes (Table 2). The *tnfxB1-tmexCD1-toprJ1* cluster was flanked by two mobile element proteins. A transposase, TnpA, is located upstream of this cluster, indicating that *tnfxB1-tmexCD1-toprJ1* might transfer from other antibiotic resistance plasmids by genetic recombination.

## DISCUSSION

Monitoring the distribution of *mcr* genes in the “one health” vision is important for the development of effective control measures. Since the novel *mcr* gene was identified on a nonconjugative IncFIA plasmid of a clinical Enterobacter roggenkampii isolate in 2020 in China, *mcr-10* was mainly reported on plasmids or chromosomes of Enterobacter spp. isolated from different sources, including clinical fecal samples, hospital sewage water, and animals. Both transferable and nontransferable *mcr-10*-carrying plasmids were reported previously (19, 23, 25). There is also a case of an *mcr-10*-carrying plasmid reported in Cronobacter sakazakii (24). However, *mcr-10*-harboring plasmids in K. pneumoniae and E. coli isolates were not reported in previous studies (26). This study discovered four *mcr-10*-harboring isolates distributed among three different bacterial species among the 200 *Enterobacterales* isolates recovered from chickens and humans in the same region (4/200, 2%). Kim et al. (27) reported a relatively high rate of *mcr-10* prevalence (17/3,675, 0.46%, K. pneumoniae [*n* = 1] and Enterobacter cloacae complex [*n* = 16]) in clinical carbapenem-resistant *Enterobacterales* (CRE) isolates in Seoul, Republic of Korea, which was the second-highest rate in all *mcr* variants, just behind *mcr-9*. The high isolation rate of *mcr-10* indicated a high transmission capacity of *mcr-10*, either by self-transmission or by site-specific recombination. A study has reported that the number of *mcr-10*-carrying fecal samples was more elevated in Asia than in Europe or the United States (28). However, after the exhaustive screening of *mcr-10* in the GenBank database, we reported significantly more *mcr-10*-positive strains in the United States, isolated from humans, water, and food. Though this might be due to sample bias, we demonstrated a worldwide distribution of *mcr-10* within different hosts. All these findings revealed a silent distribution of *mcr-10* in different sources. Thus, there is an urgent need for further surveillance to understand the prevalence and dissemination of *mcr-10*-positive isolates.

No genes encoding conjugation-related proteins were found in the Cronobacter sakazakii *mcr-10*-carrying plasmid pMCR10_145005. At the same time, two recombination sites were identified flanking the genetic element containing *mcr-10* and an integrase-encoding gene, suggesting that site-specific recombination mediated by an integrase of an integrative mobile element is a potential mechanism for mobilizing *mcr-10* (24). The three *mcr-10*-carrying plasmids in this study did not contain conjugation-related genes, and the plasmids were non-self-transmissible. We did not find any complete site-specific recombination sites but transposases in the *mcr-10*-harboring plasmids of this study. It is worth noting that *xerC-mcr*-*10* is quite well conservated, no matter whether it is located on a plasmid or in the genome with diverse insertion sequences (ISs) found upstream and downstream of this site, which implies that the area surrounding *xerC-mcr*-*10* is the high-frequency region for insertion of mobile genetic elements. Considering the non-self-transmissibility of most reported *mcr-10* plasmids (19, 24), these reports indicated diversified paths for *mcr-10* transfer. In the three novel *mcr-10*-carrying plasmids we discovered, pEC81-mcr10 showed few similarities with pKP46-mcr10 and pKP57-mcr10, so we speculate that the spreading process of *mcr-10* from chicken to human would go through several rounds of insertion or integration events among different types of plasmids.

The MIC assay in this study revealed that the colistin resistance mediated by the *mcr-10*-carrying plasmid caused a moderate increase in colistin MIC, which was consistent with previous reports (19, 24, 25), while EK6, the chromosomal *mcr-10*-harboring isolate, displayed high resistance to colistin with a MIC of 128 mg/mL, because of mutations in the two-component systems. *mcr-10* in KP46 and KP57 functions synergistically with *mcr-8*, resulting in relatively higher resistance to colistin than that of the single *mcr-10* carrier EC81.

We would like to emphasize that *mcr-8* was also found in the *mcr-10*-harboring MDR ST11 K. pneumoniae isolates. This is the first report of MDR ST11 K. pneumoniae harboring both non-self-transferable *mcr-10* and self-transferable *mcr-8*. MDR ST11 K. pneumoniae is the dominant clinical prevalent clone of KPC-producing K. pneumoniae in China and Asia and brings great difficulty to clinical treatment (26, 29). Both KP46 and KP57 are isolates with not only multiple resistance genes but also multiple virulence genes. An efficient enterobactin system and other known virulence factors indicate a potential threat to public health. Such strains may spread and evolve into MDR hypervirulent strains that cause severe and untreatable invasive infections in the clinic. Therefore, close surveillance is urgently needed to monitor the prevalence of *mcr* genes, especially in ST11 carbapenem-resistant hypermucoviscous K. pneumoniae clones in the clinical setting.

In conclusion, to our knowledge, this is the first detailed report of *mcr-10-*harboring plasmids from K. pneumoniae and E. coli and various *mcr-10-*harboring *Enterobacterales* in a “one health” approach. Extensive screening of *mcr-10*-carrying isolates in the NCBI database in this study indicated a sporadic distribution of *mcr-10* all around the world and from a variety of sources, including humans, environments, and animals, which confirms that *mcr-10* has spread among various hosts and warrants close monitoring and further studies.

## MATERIALS AND METHODS

### Bacterial isolation, detection of *mcr-10*, and antimicrobial susceptibility testing.

A surveillance study of *mcr*-harboring *Enterobacterales* recovered from feces of chickens, slaughterhouse workers, and healthy people nearby was conducted in 2019. This study was approved by the Ethical Committee of the First Affiliated Hospital of Zhejiang University with waiver of informed consent. Fecal samples (≈1.0 g) were diluted in 5 mL of sterile Luria-Bertani broth and cultured overnight at 37°C. The cultures grown overnight were plated on MacConkey agar for 18 to 24 h at 37°C. Colonies with different morphologies were repeatedly streaked on MacConkey agar to obtain pure isolates. Bacterial species were identified by using the Vitek MS and matrix-assisted laser desorption ionization–time of flight (MALDI-TOF). All isolated *Enterobacterales* were subjected to PCR and Sanger sequencing on the *mcr*-*10* gene, using primers *mcr*_10-F, GGACCGACCTATTACCAGCG, and *mcr*_10-R, GGCATTATGCTGCAGACACG (25).

Agar or broth microdilution methods evaluated antimicrobial susceptibility according to Clinical and Laboratory Standards Institute (CLSI) guidelines. The MICs of polymyxins (colistin and polymyxin B) and tigecycline were determined by the broth dilution method, and MICs of the other antibiotics were determined by the agar dilution method. Polymyxin and tigecycline resistances were defined according to clinical breakpoints of the European Committee on Antimicrobial Susceptibility Testing (EUCAST) (version 10.0) (https://eucast.org/clinical_breakpoints/), and the others were interpreted according to CLSI guidelines. Escherichia coli ATCC 25922 was used as a quality control standard for antimicrobial susceptibility testing.

### WGS and bioinformatic analysis.

Genomic DNA of *mcr-10-*positive isolates was extracted using the Gentra Puregene Yeast/Bact kit (Qiagen, CA, USA), subjected to whole-genome sequencing (WGS) on a Nanopore PromethION platform (Nanopore, Oxford, UK) following a 10-kbp library protocol, and checked with the Illumina Novaseq 6000 system (Illumina, San Diego, CA, USA), using paired-end libraries. The hybrid assembly of short Illumina reads and long PromethION reads was performed using Unicycler v0.4.8. PCR, and sequencing confirmed plasmid circularity. The annotation of the WGS data was performed by Prokka v1.17.

Multilocus sequence typing (MLST) was performed and antimicrobial resistance genes were identified using both BacWGSTdb 2.0 (30) and the Center for Genomic Epidemiology (CGE) platform. Plasmid incompatibility type was determined by PlasmidFinder 2.0. Circular comparisons between multiple genomes and plasmids were prepared using BLAST Ring Image Generator (BRIG). Linear comparisons of multiple plasmids were generated using Easyfig.2. The phylogenetic tree was generated using Prokka and Roary. The presence of strains harboring *mcr-10* was screened by performing a BLAST search on *mcr-10.1* (GenBank accession number MN179494.1) in the NCBI nr database. We screened for the presence of *mcr-10* in sequences, including complete, draft, or raw-read genome sequences deposited in GenBank, by BLAST search (https://blast.ncbi.nlm.nih.gov/Blast.cgi, accessed 26 September 2021). Matches with >90% identity and >90% coverage were retrieved from GenBank.

### Conjugation and electroporation experiments.

Conjugation assays were mainly performed according to a method described previously (31). *mcr-10*-harboring isolates were used as the donor, while E. coli J53 (sodium azide resistant) served as the recipient strain. Transconjugants were selected on Mueller-Hinton agar supplemented with sodium azide (100 mg/L) and colistin (1 mg/L). PCR and DNA sequencing were used to detect the presence of the *mcr-10* gene in transconjugants.

### S1-PFGE and Southern hybridization.

To estimate the sizes of *mcr*-positive plasmids, S1-PFGE and Southern hybridization were performed. Briefly, bacterial whole-cell DNA of *mcr*-positive isolates and their transconjugants was prepared in agarose plugs and digested with S1 nuclease (TaKaRa, Dalian, China). The DNA was separated using the CHEF-Mapper PFGE system (Bio-Rad) under the following conditions: 14°C, 6 V/cm, and a 120° pulse angle for 16 h, with the initial and final pulses conducted for 2.16 and 63.8 s, respectively. The separated DNA fragments were transferred to nylon membranes, hybridized with digoxigenin-labeled *mcr-10*- or *mcr-8*-specific probes, and detected using the nitroblue tetrazolium–5-bromo-4-chloro-3-indolylphosphate (NBT-BCIP) color detection kit (Roche, catalog no. 11745832910).

### Measurement of virulence.

The virulence of these isolates was estimated by the anticomplement killing test and Galleria mellonella infection experiment. For the anticomplement killing test, serum was collected from healthy volunteers, centrifuged to obtain normal serum, and placed in a water bath at 56°C for 30 min to inactivate complement, generating inactive serum. One hundred eighty microliters of serum was separately mixed with 20 μL diluted bacterial suspension (2 × 10^6^ CFU/mL) and incubated at 37°C for 1 h. Samples were diluted 100-fold, spread onto plates, and incubated overnight, and colonies were counted. The bacterial survival rate was calculated using the following formula: bacterial survival rate = (number of colonies with normal serum/number of colonies with inactivated serum) × 100%. For the G. mellonella infection experiment, the overnight bacterial culture was diluted to a cell density of 1 × 10^6^ CFU/mL, and G. mellonella individuals weighing ∼250 mg were randomly divided into groups with 25 individuals in each group. Each individual was injected with 20 μL of bacterial suspension, incubated at 37°C, and assessed once every 8 h for 7 days. The Kaplan-Meier estimator method was used to plot a survival curve for G. mellonella. Phosphate-buffered saline (PBS) served as the negative control. All experiments were done in triplicate.

### Data availability.

WGS data for KP46, KP57, EC81, and EK6 have been deposited in GenBank under accession no. CP088120 to CP088125, CP088126 to CP088130, CP088131 to CP088133, and CP088119, respectively.
