# Supplementary material for: Emergence of Colistin Resistance Gene mcr-10 in Enterobacterales Isolates Recovered from Fecal Samples of Chickens, Slaughterhouse Workers, and a Nearby Resident
Source: Microbiol Spectr. 2022 Apr 12;10(2):e00418-22. doi: 10.1128/spectrum.00418-22 (PMC9045214; doi:10.1128/spectrum.00418-22)
Supplement: SUPPLEMENTAL FILE 1 — Supplemental material. Download spectrum.00418-22-s0001.pdf, PDF file, 0.8 MB [file spectrum.00418-22-s0001.pdf]

Supplementary Materials

Emergence of colistin resistance gene *mcr*-10 in Enterobacterales recovered from fecal samples of chicken, slaughterhouse workers and nearby resident

Linna Xu[1,2#], Fen Wan[2#], Hao Fu[1], Biao Tang[3], Zhi Ruan[4], Yonghong Xiao[1,5], Qixia Luo[1,5*]

[1]State Key Laboratory for Diagnosis and Treatment of Infectious Diseases; Collaborative Innovation Center for Diagnosis and Treatment of Infectious Diseases, the First Affiliated Hospital of Medical School, College of medicine, Zhejiang University, Hangzhou, China.

[2]School of Laboratory Medicine and Biotechnology, Hangzhou Medical College, Hangzhou, China.

[3]Institute of Agro-product Safety and Nutrition; Zhejiang Academy of Agricultural Sciences, Hangzhou, China.

[4]Department of Clinical Laboratory, Sir Run Run Shaw Hospital, Zhejiang University School of Medicine, Hangzhou, China.

[5]Jinan Microecological Biomedicine Shandong Laboratory, Jinan, China.

***Corresponding author**

Address: 79 Qingchun Road, 310003, Hangzhou, China.

Telephone: +86-571-87236756

Fax: +86-571-87236756

E-mail addresses: qixia_luo@zju.edu.cn

[#] These authors contribute equally.

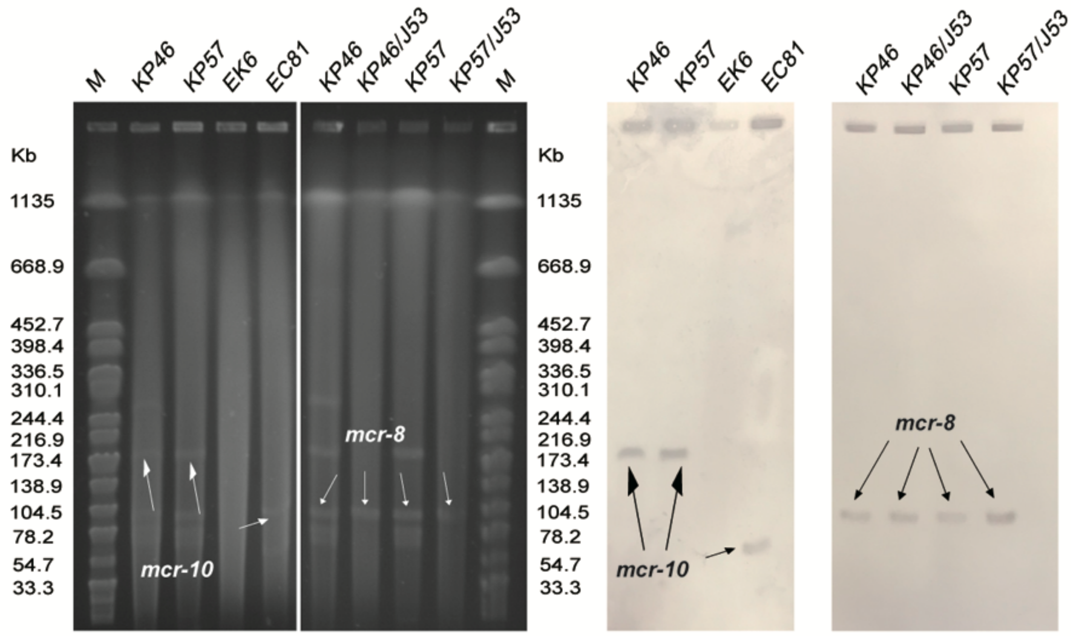

Supplementary Figure 1. S1-PFGE profiles (left) and Southern blot (right) analysis with an *mcr-10* or *mcr-8* specific probe of *mcr-10* or *mcr-8* harboring isolates, with *Salmonella enterica* serovar Branderup as a molecular mass marker. The names of the isolates are shown in the first line. The arrows in indicated the locations of *mcr-10* or *mcr-8* harboring plasmids according to the Southern blot experiment.

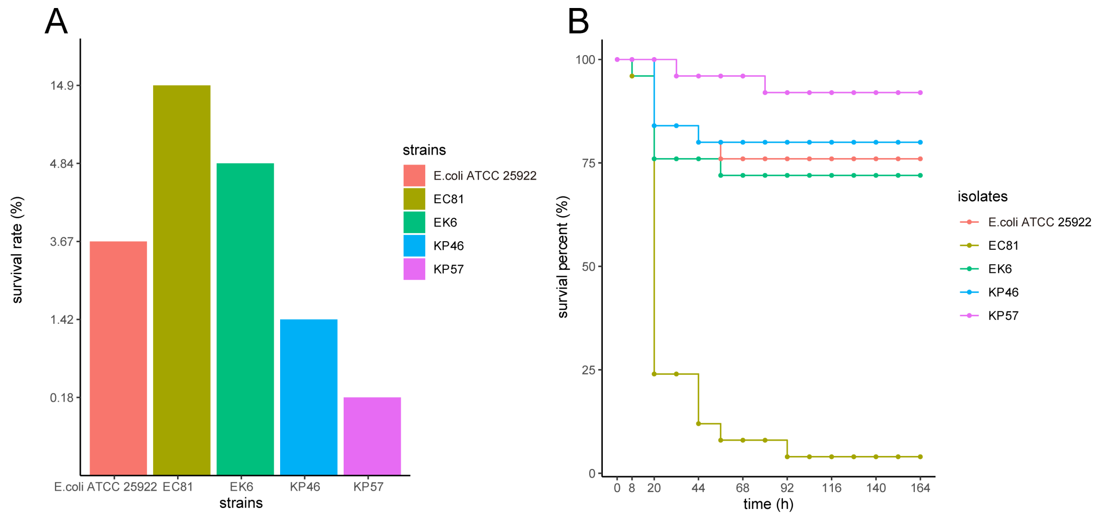

Supplementary Figure 2. Anti-complement killing test and *Galleria mellonella* infection experiment to measure virulence of the isolates in this study. A. The anti-complement killing experiment of the four *mcr-10* positive strains in this study. The ability of anti-complement killing of these strains is EC81 stronger than EK6, *E. coli* ATCC 25922, KP46, and KP57 in turn. B. The *Galleria mellonella* experiment of the four *mcr-10* positive strains in this study. The virulence is also EC81 higher than EK6, *E. coli* ATCC 25922, KP46, and KP57 in turn.

Supplementary Table 1. Strains harbouring *mcr-10* in GenBank.

Scientific name	Strain	Host Disease	WGS Accession/Run/Assembly	Host	Collection Date (year)	Location	Source
Kluyvera ascorbata	NA	NA	CP016762.1	Human	2014	Spain	Gut
Kluyvera ascorbata	NA	NA	DACSXO000000000.1	Human	2014	Spain	Gut
Kluyvera ascorbata	NA	NA	DACSXG000000000.1	Human	2014	Spain	Gut
Citrobacter freundii	B38	leg ulcer	CP016762-CP016765	Human	1998	China: Guangzhou	NA
Citrobacter freundii	strain 105	not collected	DACXTD000000000.1	Human	2015	Singapore	NA
Cronobacter sakazakii	145005	NA	JABTXY000000000.1	Human	2019	China: Chengdu, Sichuan	Stool
Enterobacter sp.	MGH 25	NA	AYJF00000000.1	Human	2013	NA	Urine
Enterobacter kobei	ECC1097	NA	NEEU00000000.1	Human	2010	China: Hangzhou	Urine
Enterobacter kobei	ECC1752	NA	NEET00000000.1	Human	2017	China: Hangzhou	NA
Enterobacter cloacae subsp. cloacae	SMART_313	clinical or host-associated bacteria	LPQI00000000.1	Human	2010	Viet Nam	NA
Enterobacter cloacae	PIMB10EC27	urinary tract infection	ASM298219v1	Human	2010	Viet Nam	Urine
Enterobacter kobei	N17-01044	NA	DACOJZ000000000.1	water	2017	Canada: Toronto	Drain
Enterobacter kobei	N17-01045	NA	DACOJY000000000.1	water	2017	Canada: Toronto	Drain
Enterobacter roggenkampii	UCI 39	NA	JCKQ00000000.1	Human	2014	USA: Irvine, CA	Urine
Enterobacter kobei	BIDMC 67	NA	JMUQ00000000.1	Human	2013	USA: Boston	Abscess
Enterobacter roggenkampii	GN02570	NA	LEES00000000.1	Human	2007	USA	NA
Enterobacter kobei	GN02825	NA	LEDC00000000.1	Human	2009	USA	Bodily fluid
Enterobacter kobei	GN02204	NA	LEEQ00000000.1	Human	2003	USA	NA
Enterobacter roggenkampii	1000_ECLO	NA	JWGU00000000.1	Human	2014	USA: WA	NA
Enterobacter roggenkampii	1001_ECLO	NA	JWGT00000000.1	Human	2014	USA: WA	NA

Enterobacter kobei	1323_ECLO	NA	JVTR00000000.1	Human	2014	USA: WA	NA
Enterobacter asburiae	C7	Cystic fibrosis	LDVX00000000.1	Human	2003	Australia: Brisbane	Lungs
Enterobacter roggenkampii	GN05753	Bloodstream infection	LRCX00000000.1	Human	2013	USA	NA
Enterobacter cloacae	CAV1757	NA	SRR2965625	Human	2012	USA:Virginia	Perirectal
Enterobacter roggenkampii	NA	NA	AAZMGS000000000.1	Human	2017	USA:Boston	Bronchial alveolar lavage
Enterobacter roggenkampii	MGH132	NA	NGRL00000000.1	Human	2015	USA: Massachusetts, Boston	NA
Enterobacter cloacae	Pasteur MLST 125	NA	SRR5666422	Human	2015	USA	Urine
Enterobacter cloacae	Pasteur MLST 41	NA	SRR5666548	Human	2015	USA	Urine
Enterobacter kobei	49530189	bacteraemia	NJCZ00000000.1	Human	2017	Australia: Adelaide	Blood culture
Enterobacter asburiae	NA	NA	AAXLNU000000000.1	Human	2017	USA:Boston	Bronchial washings
Enterobacter cloacae	HE-CRE155	NA	DRR061426	Human	2014	USA	NA
Enterobacter kobei	149G8	Not available	QMCJ00000000.1	Human	2017	France	Urine
Enterobacter kobei	CRE71	bacterial infectious disease	PXKA00000000.1	Human	2016	USA: Palo Alto, California	Bloodstream
Enterobacter kobei	CRE54	bacterial infectious disease	PXKD00000000.1	Human	2016	USA: Palo Alto, California	Bloodstream
Enterobacter cloacae	ntmc-TH	NA	BHEB00000000.1	Human	2017		NA
Enterobacter cloacae	NA	Colonization	SRR6892679	Human	2016	USA: New York City, NY	Stool

Enterobacter cloacae	strain 153	not collected	SRR4302144	Human	2014	Singapore	NA
Enterobacter cloacae	strain 123	not collected	SRR4302223	Human	2015	Singapore	NA
Enterobacter cloacae	GEO_23_Down_A	NA	QKPI00000000.1	Water	2017	USA	Water
Enterobacter asburiae	NA	NA	DACOOP000000000.1	NA	2017	NA	NA
Enterobacter asburiae	4928STDY7071310	NA	CABGKT000000000.1	Human	2018	United Kingdom	Faecal
Enterobacter roggenkampii	P088E	NA	QFVG00000000.1	Human	2016	United Kingdom: Norwich	Faecal
Enterobacter roggenkampii	WCHER090065	NA	CP017184-CP017185	Human	2016	China: Chengdu, Sichuan	NA
Enterobacter kobei	NA	NA	SRR10377181	Human	2016	USA	Urine
Enterobacter asburiae	NA	NA	ERR3567685	NA	2019	NA	NA
Enterobacter cloacae	NA	NA	DRR178362	Human	2017	Japan	NA
Enterobacter cloacae	1476	Urinary tract infection 33	JAAATK000000000.1	Human	2015	Nigeria	Urine
Enterobacter cloacae	NA	NA	ERR3712381	NA	2019	Netherlands	NA
Enterobacter cloacae	NA	NA	ERR3712412	NA	2019	Netherlands	NA
Enterobacter cloacae	NA	NA	ERR3712576	NA	2019	Netherlands	NA
Enterobacter asburiae	AS012371	Pneumonia	VKVT00000000.1	Human	2016	USA	Lung
Enterobacter roggenkampii	AS012313	Pneumonia	VLNB00000000.1	Human	2015	USA	Lung
Enterobacter roggenkampii	AS012293	Pneumonia	VLND00000000.1	Human	2015	USA	Lung
Enterobacter hormaechei	CVM 45669	NA	ABANSR000000000.1	Environmental	2019	USA:KS	Environmental
Enterobacter cloacae	2019GO-00051	NA	AAXLNL000000000.1	Human	2019	USA	NA
Enterobacter cloacae	NA	NA	SRR6676725	Human	2015	USA: Michigan	Urine

Enterobacter cloacae	NA	NA	SRR6676731	Human	2014	USA: Michigan	Blood
Enterobacter cloacae	NA	NA	SRR6676733	Human	2014	USA: Michigan	Blood
Enterobacter cloacae	NA	NA	SRR6676744	Human	2013	USA: Michigan	Blood
Enterobacter cloacae	N17-01386	NA	DACOKA000000000.1	Water	2017	Canada: Toronto	Drain
Enterobacter cloacae	microbial	NA	SRR12279266	Human	2020	USA	Urine
Enterobacter roggenkampii	YK16	NA	JABJWE000000000.1	Chicken	2019	China: Beijing	NA
Enterobacter cloacae	RHBSTW-00399	NA	CP009756	Water	2017	United Kingdom	Wastewater effluent sample
Enterobacter asburiae	RHBSTW-01009	NA	SRS6792410	Water	2017	United Kingdom	Wastewater effluent sample
Enterobacter cloacae	NA	not provided	SRR12352166	Human	2018	USA	Tracheal aspirate
Enterobacter roggenkampii	Ed-982	NA	CP060733-CP060736	Water	2019	China: Guangdong	Sewage water
Enterobacter roggenkampii	NA	NA	CP060737- CP060741	Water	2019	China: Guangdong	Sewage water
Enterobacter roggenkampii	Ecl_20_981	NA	CP048650-CP048653	Water	2019	China: Shenzhen	Medical waste water
Enterobacter cloacae	Enterobacter cloacae ARLG-4177	NA	SRR12508629	Human	2017	USA: South	Urine
Enterobacter asburiae	Enterobacter asburiae ARLG-4178	NA	SRR12508786	Human	2017	USA: Midwest	Urine

Enterobacter cloacae	Enterobacter cloacae ARLG-3588	NA	SRR12509054	Human	2016	USA: Northeast	Urine
Enterobacter cloacae	Enterobacter cloacae ARLG-3552	NA	SRR12509102	Human	2016	USA: Northeast	Urine
Enterobacter kobei	MGH240	NA	JACRQY000000000.1	Human	NA	USA: MA, Boston	NA
Enterobacter cloacae	CAVp273	NA	SRR6061983	Human	2015	USA:Virginia	Peri-rectal
Enterobacter cloacae	E64	NA	SRR9696912	Water	2014	Australia: Sydney	ARJO bathroom
Enterobacter kobei	STW0522-51	NA	DRR199696	Water	2018	Japan:Tokyo	Hospital sewage tank water
Enterobacter roggenkampii	STW0522-66	NA	DRR199711	Water	2018	Japan:Tokyo	Hospital sewage tank water
Enterobacter kobei	U119_10	NA	JAANZQ000000000.1	Water	2018	South Africa:KwaZulu-Natal	wastewater
Enterobacter cloacae	2020HL-00857	NA	SRR13183123	Human	2020	USA	Urine
Enterobacter ludwigii	EC56	bacteremia	JADRHY000000000.1	Human	2018	Japan	Blood
Enterobacter cloacae	2016CRE_23	mass screening	SRR13077397	Human	2016	Singapore	NA
Enterobacter roggenkampii	H06	NA	JAEOAW000000000.1	Food	2018	USA:Washington, DC	Ground beef
Enterobacter roggenkampii	OIPH-N260	NA	AP023447-AP023451	Human	2019	Japan:Osaka	NA
Enterobacter cloacae	NA	NA	ERR4977469	NA	2020	Netherlands	NA

Enterobacter cloacae	NA	NA	ERR4977491	NA	2020	Netherlands	NA
Enterobacter cloacae	NA	NA	ERR4977514	NA	2020	Netherlands	NA
Enterobacter cloacae	NA	NA	ERR4977529	NA	2020	Netherlands	NA
Enterobacter roggenkampii	97COLEN	NA	JAFGZI000000000.1	Human	2019	Singapore	Faecal
Enterobacter cloacae	En37	NA	DRX274627	Dog	2015	Japan:Chiba	NA
Enterobacter kobei	12982	NA	JAGFWV000000000.1	Human	2012	China	Sputum
Enterobacter kobei	11778	NA	JAGFWW000000000.1	Human	2011	China	Secretion
Enterobacter ludwigii	11894	NA	JAGFWU000000000.1	Human	2011	China	Throat swab
Enterobacter cloacae	Not collected	Bloodstream infection	SRR14209343	Human	2018	Singapore	Blood
Enterobacter cloacae	Survcare369	not determined	JAFHGL000000000.1	Human	2019	Germany	Rectal swab/stool
Enterobacter kobei	sc19427838	not determined	JAFHQH000000000.1	Human	2019	Germany	Rectal swab/stool
Enterobacter roggenkampii	120063	NA	JAHETR000000000.1	Human	2019	China: Chengdu, Sichuan	Blood
Enterobacter roggenkampii	120088	NA	JAHEUJ000000000.1	Human	2019	China: Chengdu, Sichuan	Blood
Enterobacter cloacae	NA	not collected	SRR14808320	Human	2013	Canada: Quebec	Respiratory system
Enterobacter cloacae	NA	not collected	SRR14810469	Human	2014	Canada: Ontario	Blood
Enterobacter cloacae	NA	not collected	SRR14917121	Human	2018	Canada: Ontario	Respiratory system
Enterobacter cloacae	2021DK-00109	NA	SRR15167009	Human	2021	USA	Bile
Enterobacter roggenkampii	CORE_Ent4	urinary tract	JAFFSN000000000.1	Human	2016	USA: Michigan	Urine

		infection					
Enterobacter roggenkampii	CORE_Ent3	pneumonia	JAFFSO000000000.1	Human	2017	USA: Michigan	Sputum
Enterobacter roggenkampii	ECC275	NA	JAHZRB000000000.1	Human	2018	China: Shenzhen	Shunt fluid
Enterobacter hormaechei	ECC59	NA	CP080470-CP080477	Human	2017	China: Guangdong	Broncho-alveolar lavage
Enterobacter sichuanensis	ME2L-20-110	NA	JABUMZ000000000.1	Water	2016	USA:Ohio	Sewage
Enterobacter hormaechei	WHC182	NA	JAINDC000000000.1	Human	2020	China: Changsha	NA
Enterobacter roggenkampii	Res13-Abat-PEA21-P1-02-B	NA	JADAJI000000000.1	Pig	2018	Canada: Quebec	Swab
Enterobacter roggenkampii	Res13-Abat-PEA21-P1-01	NA	JADAJH000000000.1	Pig	2018	Canada: Quebec	Swab
Klebsiella pneumoniae	strain 58	not collected	SRR4302109	Human	2014	Singapore	NA
Klebsiella pneumoniae	strain 60	not collected	SRR4302112	Human	2014	Singapore	NA
Klebsiella pneumoniae	GEO_33_Down_A	NA	QKPD00000000.1	Water	2017	USA	Water
Klebsiella pneumoniae	NA	NA	ERR2497302	Human	2017	Malawi	NA
Klebsiella pneumoniae	TUM14004	NA	BIGJ00000000.1	Human	2013	Japan	NA
Klebsiella pneumoniae	NA	NA	ERR4372136	Human	2016	United Kingdom	Rectal Screen
Klebsiella quasipneumoniae	NA	NA	ERR4968650	NA	2020	NA	NA
Escherichia coli	AZ74	NA	LLYM00000000.1	Human	2014	China:Heilongjiang	Gut
Escherichia coli	KFu029	NA	DRR063351	Water	NA	Japan	Water
Escherichia coli	24.1-R2	NA	MOYV00000000.1	Human	2012	Australia: Sydney	Faecal
Escherichia coli	19NC09CB16_EC	NA	AASPCH000000000.1	Food	2019	USA:NC	Chicken Breast

							(Gallus gallus)
Escherichia coli	VREC0181	NA	DADKVK000000000.1	Environmental	2015	NA	NA
Escherichia coli	ST-57-13	NA	DADRWT000000000.1	Water	2018	Australia	septic tank
Escherichia coli	PNUSAE065888	NA	AAYBHK000000000.1	Human	NA		NA
Escherichia coli	1187049	Not available	ABATJF000000000.1	Human	2021	United Kingdom: United Kingdom	NA
Raoultella ornithinolytica	FDAARGOS_431	Screening	CP046672	Human	2015	Canada:BC	Rectal
Raoultella ornithinolytica	TUM14061	NA	BIIO00000000.1	Human	2013	Japan	NA
Raoultella ornithinolytica	NUITM-VR1	NA	CP046672	NA	2021	Viet Nam:Hanoi	NA
Klebsiella pneumoniae	INF093-sc-2280036	NA	ERR1008652	Human	2015	Australia	NA
Klebsiella pneumoniae	INF094-sc-2280038	NA	ERR1008653	Human	2015	Australia	NA
Klebsiella pneumoniae	INF133-sc-2279960	NA	ERS710862	NA	2015	NA	NA
Klebsiella pneumoniae	strain 57	not collected	SRR4302108	Human	2015	Singapore	NA
Klebsiella pneumoniae	strain 59	not collected	SRR4302110	Human	2014	Singapore	NA
Klebsiella quasipneumoniae subsp. quasipneumoniae	NA	NA	SRR9217808	Human	2014	Singapore	Stool/Rectal Swab
Klebsiella quasipneumoniae subsp. quasipneumoniae	NA	NA	SRR9217836	Human	2014	Singapore	Rectal swab
Klebsiella quasipneumoniae subsp. quasipneumoniae	NA	NA	SRR9217837	Human	2014	Singapore	Rectal swab

Klebsiella pneumoniae	MyNCGM084	NA	DRR198022	Human	2016	Myanmar:Naypyitaw	NA
Klebsiella pneumoniae	MyNCGM088	NA	DRR198023	Human	2016	Myanmar:Naypyitaw	NA
Klebsiella pneumoniae	NA	NA	ERR4783007	NA	2020	Nigeria	NA
Klebsiella quasipneumoniae	NA	NA	ERR4783313	NA	2020	NA	NA
Klebsiella quasipneumoniae	NA	NA	ERR4783439	NA	2020	NA	NA
Klebsiella pneumoniae	NA	asymptomatic	SRR10005937	Human	2015	Laos:Vientiane	Stool
Klebsiella pneumoniae	NA	asymptomatic	SRR10006013	Human	2015	Laos:Vientiane	Stool
Escherichia coli	NA	NA	SRR11046325	Water	2017	United Kingdom: Oxford	Sink drain aspirate
Escherichia coli	NA	NA	SRR11046541	Water	2017	United Kingdom: Oxford	Sink drain aspirate
Escherichia coli	NA	NA	SRR11046574	Water	2017	United Kingdom: Oxford	Sink drain aspirate
Escherichia coli	NA	NA	SRR11046575	Water	2017	United Kingdom: Oxford	Sink drain aspirate
Escherichia coli	NA	NA	SRR11046670	Water	2017	United Kingdom: Oxford	Sink drain aspirate
Escherichia coli	NA	NA	DABWMN000000000.1	Human	2013	Spain	Human gut
Escherichia coli	NA	NA	DABWMK000000000.1	Human	2013	Spain	Human gut
Escherichia coli	NA	NA	SRR11046508	Water	2017	United Kingdom: Oxford	Sink drain aspirate
Escherichia coli	NA	NA	SRR11046609	Water	2017	United Kingdom: Oxford	Sink drain aspirate

Supplementary Table 2. *mcr-10*-harbouring plasmids from GenBank and this study.

Plasmid	Accession no.	Species, strain	Source	Year	Country	Plasmid replicon	Size
pMCR10_090065	CP045065	Enterobacter roggenkampii 090065	Human ascites	2016	China	FIA	71,775 bp
pOZ172	CP016763	Citrobacter freundii B38	Human leg ulcer	1998	China	FIB, FII	127,005 bp
pEC27-2	CP020091	Enterobacter cloacae PIMB10EC27	Human urine	2010	Vietnam	FII	84,602 bp
unnamed1	CP023893	Raoultella ornithinolytica FDAARGOS_431	Human rectal swab	2015	Canada	FIB, FII	231,294 bp
pYK16-mcr-10	MT468575	Enterobacter roggenkampii YK16	Chicken	2019	China	FIA	117,855 bp
pEN37S	AP024497	Enterobacter cloacae En37	Dog	2015	Japan	FIB	70,277 bp
pECL981-1	CP048651	Enterobacter roggenkampii	Hospital water	2020	China	FIB-FII	161,986 bp
pECL983-1	CP060738	Enterobacter roggenkampii	Hospital water	2020	China	FIB	100,102 bp
pMCR10_145005	JABTXY010000030	Cronobacter sakazakii 145005	Human fecal	2019	China	FIB	120,500 bp
plasmid 5	LR890193.1	Klebsiella pneumoniae INF133-sc-2279960	Hospital	2015	Australia	FIIK	120,029 bp
pKP46-mcr10	CP088121	Klebsiella pneumoniae KP46	Chicken	2019	China	FIB-FII	185,056 bp
pKP57-mcr10	CP088127	Klebsiella pneumoniae KP57	Chicken	2019	China	FIB-FII	186,040 bp
pEC81-mcr10	CP088132	Escherichia coli EC81	Human fecal-farmer	2019	China	FIA	62,662 bp
